# Diabetes changes the levels of ionotropic glutamate receptors in the rat retina

**Published:** 2009-08-17

**Authors:** Ana R. Santiago, Joana M. Gaspar, Filipa I. Baptista, Armando J. Cristóvão, Paulo F. Santos, Willem Kamphuis, António F. Ambrósio

**Affiliations:** 1 Center for Neuroscience and Cell Biology, Department of Zoology, University of Coimbra, Coimbra, Portugal; 2 Center of Ophthalmology and Vision Sciences, Institute of Biomedical Research on Light and Image (IBILI), Faculty of Medicine, University of Coimbra, Coimbra, Portugal; 3 Department of Zoology, University of Coimbra, Portugal; 4 Netherlands Institute for Neuroscience (NIN)-KNAW, Department of Astrocyte Biology and Neurodegeneration, Amsterdam, The Netherlands

## Abstract

**Purpose:**

Diabetic retinopathy (DR) is a leading cause of vision loss and blindness among adults between the age 20 to 74. Changes in ionotropic glutamate receptor subunit composition can affect retinal glutamatergic neurotransmission and, therefore, contribute to visual impairment. The purpose of this study was to investigate whether diabetes leads to changes in ionotropic glutamate receptor subunit expression at the protein and mRNA level in the rat retina.

**Methods:**

Changes in the expression of ionotropic glutamate receptor subunits were investigated at the mRNA and protein levels in retinas of streptozotocin (STZ)-induced diabetic and age-matched control rats. Animals were euthanized one, four and 12 weeks after the onset of diabetes. Retinal protein extracts were prepared, and the receptor subunit levels were assessed by western blotting. Transcript levels were assessed by real-time quantitative PCR.

**Results:**

Transcript levels of most ionotropic glutamate receptor subunits were not significantly changed in the retinas of diabetic rats, as compared to age-matched controls but protein levels of α-amino-3-hydroxyl-5-methyl-4-isoxazole-propionate (AMPA), kainate, and N-methyl-D-aspartic acid receptors (NMDA) receptors were found to be altered.

**Conclusions:**

The results provide evidence that diabetes affects the retinal content of ionotropic glutamate receptor subunits at the protein level. The possible implications of these changes on retinal physiology and visual impairment in DR are discussed.

## Introduction

Diabetic retinopathy (DR) is a leading cause of vision loss and blindness among adults in developed countries. The mechanisms by which diabetes causes vision loss are still not clearly understood. Vascular changes in DR are well documented, and include blood-retinal barrier breakdown, loss of pericytes and endothelial cells, the formation of microaneurysms and basement membrane thickening [1]. Changes in the neural retina during diabetes have also been reported [2-4]. These alterations may account for loss in contrast sensitivity and color vision and alterations in the electroretinogram [5-8].

Glutamate is the main excitatory neurotransmitter in the retina. It is required for the transmission of visual signals from the photoreceptors to the ganglion cells. Glutamate receptors are divided into two main groups: the fast-acting ligand-gated ionotropic channels and the slower-acting metabotropic receptors. The ionotropic receptors are cation-specific ion channels, and are subdivided into three groups: α-amino-3-hydroxy-5-methyl-4-isoxazolepropionate (AMPA), kainate, and *N*-methyl-d-aspartate (NMDA) receptor channels. In the mammalian retina, AMPA, kainate, and NMDA receptor subunits have been shown to have a widespread and differential distribution throughout the retina [9-14].

Several studies have indicated that diabetes induces changes in glutamate receptors. The ob/ob mouse, a model of type 2 diabetes, was found to have increased binding sites for NMDA and AMPA receptors in the gray matter of the spinal cord [15]. In the brain of streptozotocin (STZ)-induced diabetic rats, the binding properties of AMPA receptors and the expression of GluR1 subunit are decreased [16], without changes in the transcript levels of GluR1, GluR2/3, NR1, and NR2A subunits after three months of diabetes [17]. In the hippocampus, immunoreactivity for NR2A and NR2B subunits are reduced in diabetic rats. These changes are prevented by insulin treatment [18,19]. Additionally, glutamatergic dysfunction in the hippocampus has been suggested to be associated with cognitive impairment in the STZ-induced diabetic rat [20]. Moreover, it was reported that the transcript levels for GluR1, GluR2, GluR3, NR2A, and NR2B subunits are significantly upregulated in the dorsal horn of the spinal cord in STZ-induced diabetic rats [21].

Overactivation of glutamate receptors is considered to be potentially involved in neurodegeneration in some retinal diseases, such as glaucoma and retinitis pigmentosa [22,23], and it may be also implicated in retinal neurodegeneration during diabetes. In the retina, it was shown that glutamate metabolism and concentration are altered after short-term experimental diabetes. Diabetic rat retinas are less able to convert glutamate into glutamine [24] and have higher levels of glutamate [25], probably explaining increased vitreous glutamate concentration observed in patients with proliferative DR [26]. Moreover, the high-affinity L-glutamate/L-aspartate transporter (GLAST) is impaired in retinal Müller cells isolated from STZ-induced diabetic rats, probably due to oxidation of the glutamate transporter [27]. Previously, we demonstrated that diabetes increases the evoked release of d-aspartate in the retina [28], suggesting that glutamatergic neurotransmission can be affected in the retinas of diabetic rats. Moreover, we showed that elevated glucose concentration changes the protein levels of GluR1, GluR2, GluR6/7, and KA2 subunits in cultured rat retinal neural cells [29], and more recently, we have demonstrated, in postmortem human retinas, that there are alterations in the levels of ionotropic glutamate receptor subunits in diabetic patients [30]. It was also demonstrated an increase in NR1 and GluR2/3 immunoreactivities in ganglion, amacrine, and bipolar cells in rats after 4 and 16 weeks of diabetes [31].

Taken together, these results support a role for glutamate in the pathogenesis of DR, suggesting that it may be involved in retinal neural dysfunction during diabetes. Changes in the composition of ionotropic glutamate receptor subunits may alter the properties of ionotropic glutamate receptors, and consequently may change the glutamatergic neurotransmission in the retina, thus contributing to visual impairment. In addition, those changes may alter the vulnerability of retinal neurons to neurodegeneration. Thus, the aim of this work was to investigate whether diabetes changes ionotropic glutamate receptor subunits in the retina at mRNA and protein levels.

## Methods

### Animals

All procedures involving animals were conducted in accordance with the Association for Research in Vision and Ophthalmology (ARVO) statement for the use of animals in ophthalmic and vision research. Two-month-old Wistar rats purchased from Charles River (Barcelona, Spain) were housed under a 12 h light/12 h dark cycle with standard chow and water ad libitum. Animals were randomly assigned to control or diabetic groups. Diabetes was induced with an intraperitoneal injection of 65 mg/kg STZ (Sigma, St. Louis, MO), dissolved in citrate buffer, pH 4.5. Two days post-STZ injection, hyperglycemia was confirmed by blood glucose exceeding 250 mg/dl with Ascensia Elite (Bayer, Portugal). Diabetic rats and age-matched controls were euthanized under deep anesthesia followed by decapitation, one, four, and 12 weeks after the onset of diabetes. Before sacrifice, rats were weighed and blood glucose was measured.

### Sample preparation for western blot analysis

Each sample was composed of both retinas from the same animal (n=number of animals). The animals were euthanized, the eyes were enucleated, and the retinas were dissected in cold phosphate-buffered saline (PBS) that contained 137 mM NaCl, 2.7 mM KCl, 10 mM Na_2_HPO_4_, 1.8 mM KH_2_PO_4_, pH 7.4. The retinas were then homogenized in 20 mM Tris-HCl, 2 mM EDTA, 2 mM EGTA, pH 7.2, supplemented with complete miniprotease inhibitor cocktail tablets (Roche, Basel, Switzerland), at 4 °C. The homogenate was centrifuged at 960x g for 5 min at 4 °C. The supernatant was collected and centrifuged at 15,800x g for 20 min at 4 °C. The pellet was dissociated by sonication and resuspended in 20 mM Tris-HCl, 2 mM EDTA, 2 mM EGTA, 1% Triton X-100, 0.5% SDS, pH 7.2, supplemented with the protease inhibitor cocktail, at 4 °C. Protein concentration was determined by the Bio-Rad Bradford method, and the samples were denaturated following addition of 2X concentrated sample buffer, which contained the following: 100 mM Tris-HCl:100 mM bicine, 8 M urea, 2% SDS, 2% β-mercaptoethanol, 0.005% bromophenol blue. After heating for 5 min at 95 °C, the samples were frozen at −20 °C until western blot analysis.

### Western blot analysis

Equivalent amounts of protein (15 μg for GluR1, GluR6/7, and NR1 subunits; and 60 μg for GluR2, GluR2/3, GluR4, NR2C and NR3A subunits) were used for western blot analysis. The amounts of protein used were under the saturation limit of the system.

Proteins were separated by SDS–PAGE (7.5%), and transferred via electrophoresis to polyvinylidene fluoride (PVDF) membranes. The membranes were blocked for 1 h at room temperature in Tris-buffered saline that contained 137 mM NaCl, 20 Tris-HCl mM, pH 7.6, with 0.1% Tween-20 (TBS-T) and 5% skimmed milk. Incubation with the primary antibodies (Table 1) was performed overnight at 4 °C. After washing four times during 1 h in TBS-T in TBS-T with 0.5% skimmed milk, the membranes were incubated for 1 h at room temperature with 1:20,000 anti-rabbit IgG (GE Healthcare, Buckinghamshire, UK), an alkaline phosphatase-linked secondary antibody, in TBS-T with 1% skimmed milk. The membranes were processed for detection of ionotropic glutamate receptor subunits using the Enhanced Chemi-Fluorescence system (GE Healthcare) on a gel imager (Versa Doc Imaging System; Bio-Rad, Hercules, CA), and digital quantification of bands intensity was performed (Quantity One; Bio-Rad).

**Table 1:** Primary antibodies used in this study

**Primary antibody**	**Dilution**	**Source**
Rabbit anti-GluR1	1:500	Upstate (Lake Placid, NY)
Rabbit anti-GluR2	1:500	BD Biosciences (Heidelberg, Germany)
Rabbit anti-GluR2/3	1:500	Chemicon (Temecula, CA)
Rabbit anti-GluR4	1:500	Chemicon
Rabbit anti-GluR6/7	1:500	Upstate
Rabbit anti-NR1	1:1 200	Tocris (Ellisville, MO)
Rabbit anti-NR2C	1:1 000	BD PharMingen (San Diego, CA)
Rabbit anti-NR3A	1:1 000	Upstate

The primary antibodies were prepared in TBS-T with 1% skimmed milk, diluted according the table. The brand name of the antibody is also listed.

The membranes were then reprobed and tested for α-tubulin (Sigma-Aldrich, Lisboa, Portugal), which was used as a loading control. Briefly, the membranes were incubated for 1 h at room temperature with a 0.1 M glycine buffer (pH 7.2), blocked as previously described and then incubated with 1:3,000 mouse anti-α-tubulin antibody (Sigma). The membranes were then washed and incubated with 1:20,000 anti-mouse IgG (GE Healthcare), an alkaline phosphatise-linked secondary antibody in TBS-T with 1% skimmed milk.

### Real-time quantitative PCR

#### Isolation of total RNA from rat retinas

Each sample was composed of one retina (n=number of animals). The animals were euthanized, the eyes were enucleated, and the retinas were dissected in cold PBS and stored at −80 °C. Retinal tissue was homogenized, and total RNA was isolated by a single-step method, based on guanidine thiocyanate extraction, according to the manufacturer’s instructions (Ultraspec; Biotecx Laboratories, Inc., Houston, TX). Isolated RNA was dissolved in 16 µl diethylpyrocarbonate (DEPC)-treated water. The concentration and quality of total retinal RNA were determined (2100 Bioanalyser; Agilent Technologies Netherlands BV, Amstelveen, The Netherlands). The integrity of RNA, expressed as RNA Integrity Number (RIN) was around 9.0, indicating, high-quality, non-degraded RNA.

#### Reverse transcription

Total RNA was treated with DNase-I (1 unit DNase-I, Amplification Grade; Invitrogen BV, Breda, The Netherlands) to degrade possible genomic DNA contamination. Then, 5 μl of Dnase-I treated total RNA were reverse transcribed into first-strand cDNA with 100 U/μl of RNase H- reverse transcriptase (Superscript III, Invitrogen) and 50 ng/μl random hexamer primers, for 60 min at 50 °C. The resultant cDNA sample was diluted 1:1 with 100 mM Tris and 1 mM EDTA, and from all cDNA samples a 1:20 dilution was prepared for qPCR analysis [32]. All samples were stored at −20 °C until analysis.

Genomic DNA contamination was assessed with a conventional end point PCR for β-actin, using intron-spanning primers, under the following conditions: annealing at 55 °C, elongation at 72 °C, denaturing at 94 °C, 90 s each step for 35 cycles, with 1.5 mM Mg^2+^ and 0.75 U Taq DNA polymerase (Qiagen, Westburg, The Netherlands). The resultant PCR product was analyzed by agarose gel electrophoresis. A single band of the anticipated exon-size was found in all samples, demonstrating the absence of genomic contamination. Nontemplate and nonamplicon controls were subjected to PCR amplification, but they never yielded PCR products.

#### qPCR primers

qPCR primer pairs were designed using Primer Express V 2.0 software (PE Applied Biosystems, Warrington, UK). Details of the primers and the GenBank Accession numbers are given in Table 2.

**Table 2:** Primers for qPCR analysis.

**Gene**	**GenBank**	**UniGene name**	**UniGene symbol**	**Forward primer**	**Reverse Primer**	**bp**
*Hprt*	NM_012583	Hprt	Rn.47	ATGGGAGGCCATCACATTGT	ATGTAATCCAGCAGGTCAGCAA	77
*Ywhaz*	NM_013011	Ywhaz	Rn.1292	CAAGCATACCAAGAAGCATTTGA	GGGCCAGACCCAGTCTGA	76
*Rho*	NM_033441	Rho	Rn.92530	GCAACAGGAGTCGGCTACCA	GCATAGGGAAGCCAGCAGATC	99
*Tbp*	NM_001004198	Tbp	Rn.22712	ACCAGAACAACAGCCTTCCACCTT	TGGAGTAAGCCCTGTGCCGTAAG	116
*Ubc*	NM_017314	Ubc	Rn.3761	TCGTACCTTTCTCACCACAGTATCTAG	GAAAACTAAGACACCTCCCCATCA	82
*GluR1*	NM_031608	Gria1	Rn.29971	GAGCAACGAAAGCCCTGTGA	CCCTTGGGTGTCGCAATG	80
*GluR1 flip*	M38060			GAAGCAAGGACTCCGGAAGTAA	GTAGAACACGCCTGCCACATT	71
*GluR1 flop*	M36418			GTCCGCCCTGAGAAATCCA	AGCCCCTGCTCGTTCAGTT	57
*GluR2*	NM_017261	Gria2	Rn.91361	AACGAGTACATCGAGCAGAGGAA	GATGCCGTAGCCTTTGGAATC	78
*GluR2 short*	M38061			TTGAGTTCTGTTACAAGTCAAGGGC	AGGAAGATGGGTTAATATTCTGTGGA	81
*GluR2 long*	NM_017261			GCCTTGGTTTGGCAATGC	GACATCACTCAAGGTCATCTTCATTC	92
*GluR2 flip*	M38061			GGAACCCCAGTAAATCTTGCAGT	GAGTCCTTGGCTCCACATTCAC	107
*GluR2 flop*	M36419			CATCGCCACACCTAAAGGATC	CAATTTGTCCAACAGGCCTTGT	88
*GluR3*	NM_032990	Gria3	Rn.74049	TTCGGAAGTCCAAGGGAAAGT	CACGGCTTTCTCTGCTCAATG	76
*GluR3 flip*	M38062			GGAATGTGGAGCCAAGGACTC	GCTCAGGCTTAGAGCACTGGTC	58
*GluR3 flop*	M36420			GGCAACCCCTAAAGGCTCAG	AATACTGCCAGGTTAACAGCATTTC	51
*GluR4*	NM_017263	Gria4	Rn.10938	GGCTCGTGTCCGCAAGTC	TTCGCTGCTCAATGTATTCATTC	77
*GluR4 short*	S94371			TGATAGAGTTCTGTTACAAGTCCAGGG	CGAGGAAGTTGGGTTAAAAGTCTGT	86
*GluR4 long*	NM_017263			CCAGGGCAGAGGCGAAG	CGTTTTCTCCCACACTCCCA	93
*GluR4 flip*	M38063			TTTTGAAACTCAGTGAGGCAGG	CGTACCACCATTTGTTTTTCAGC	57
*GluR4 flop*	M36421			CCTCTTGGACAAATTGAAAAACAA	CCGCTGCCACATTCTCCTT	57
*GluR7*	NM_181373	Grik3	Rn.92477	AAGGCAAAGGAGACCCGAAAG	CATGGTTTCCCCGGTAGGTAAG	110
*NR1*	NM_017010	Grin1	Rn.9840	CTGTTCTTCCGCTCAGGCTTT	ATGAAGACCCCTGCCATGTTC	200
*NR2C*	NM_012575	Grin2c	Rn.9709	GGATCTGCCAGAACGAGAAGA	TTGTTGCCCCAGTTCTCGA	390
*NR3A*	AF073379	Grin3a	Rn.42928	CAGTCTTCGGAAACCTCATCG	TGACAGTTCTCATGCGCTTGT	63

The primers used for qPCR were as shown. The GenBank accession codes and the anticipated size of the amplified product are listed.

#### Real-time quantitative PCR

Real-time qPCR is based on the real-time monitoring of fluorescent SYBR Green I (Prism 5700; Applied Biosystems Inc., Nieuwekerk a/d IJssel, The Netherlands). The qPCR conditions were as follows: 1× SYBR Green PCR buffer; 3.5 mM MgCl_2_; 200 µM dATP, 200 μM dGTP, and 200 μM dCTP, and 400 µM dUTP; 0.5 U Taq polymerase (AmpliTaq Gold; Applied Biosystems); 0.2 U uracil-N-glycosylase (UNG; AmpErase; Applied Biosystems), 2 pmol primers; and 2 µl of the 1:20 dilution of cDNA in a total volume of 20 µl. An initial step of 50 °C for 2 min was used for UNG incubation, followed by 10 min at 95 °C to inactivate UNG and to activate the Taq polymerase. Cycling conditions were a melting step at 95 °C for 15 s and annealing–elongation at 60 °C for 1 min, for 40 cycles. The real-time detection of double-stranded DNA allows the construction of a dissociation curve at the end of the PCR run by ramping the temperature of the sample from 60 °C to 95 °C, while continuously collecting fluorescence data. The curves of the melting profiles showed a single product and did not reveal accumulation of primer dimers. Nontemplate and nonamplicon controls were included for each primer pair to check for any significant levels of contaminants, which resulted in a difference of at least five cycles of the C_t_ values compared to the template containing samples. Gel electrophoresis of amplicons showed the correct size.

### PCR amplification efficiency

In previous work, the amplification efficiency (E) of each primer pair was determined on a dilution series of cDNA. The values of E were found to be close to the optimal value of 2 for all pairs [32]. For all calculations, E=2 was used.

### Normalization

To correct for differences in cDNA load between different samples, the target PCR has to be normalized to a reference PCR. The normalization was performed as described previously in detail [32]. Using the data from all cDNA samples, for each gene the pair-wise variation in relation to all other genes was determined as the standard deviation of the logarithmically transformed expression ratios. The internal gene stability measure M was defined as the average pair-wise variation with all other control genes. Stepwise exclusion of the gene with the highest M value identified the combination of two constitutively expressed genes that have the most stable expression in the tested samples. This analysis is facilitated by the use of the virtual basic applet GeNorm developed by Vandesompele et al. [33]. In our model, ubiquitin C* (Ubc)* and TATA-box binding protein (*Tbp*) were identified as the most stable genes. Indeed, statistical analysis with Student’s *t*-test confirmed that these genes are not significantly altered by diabetes. To measure expression levels accurately, normalization to multiple reference genes is preferred. A normalization factor based on the expression levels of *Ubc* and *Tbp* was calculated by using the geometric mean of the C_t_. In addition, normalization factors were calculated for *Tbp*-*Ubc*-* Rho*, and so forth. The pair-wise variation (V) was calculated between these normalization factors; a large V value means that the added gene has a significant effect and should be included for the final normalization factor. After the suggested cut-off value of 0.15 by Vandesompele et al. [33], five genes were selected as reference genes, and their transcript levels were used to calculate the normalization factor: hypoxanthine guanine phosphoribosyl transferase (*Hprt*); tyrosine 3-monooxygenase/tryptophan 5-monooxygenase activation protein, zeta polypeptide (*Ywhaz*); rhodopsin (*Rho*); *Ubc*; and *Tbp*.

### Quantitative assessment of target gene expression

The qPCR C_t_ values were converted to “absolute amounts” (C x E^-Ct^ with C=10^10^) reflecting the amount of transcript in the cDNA sample (E^-Ct^). For normalization, the absolute amount of the target gene was divided by the normalization factor [32].

### Statistical analysis

Results are presented as mean±standard error of the mean. Statistical analysis was performed using the Student’s *t*-test. Significant difference was considered to be present for p<0.05.

## Results

### Animals

The average weight and blood glucose values for the rats at the time of death are given in Table 3. There was a significant weight loss in STZ-diabetic rats, compared with age-matched controls, at one, four, and 12 weeks after the induction of diabetes. Blood glucose levels were also significantly increased at these time points.

**Table 3:** Weight and blood glucose levels at time of death.

**Group**	**n**	**Weight (g)**	**Blood glucose (mg/dl)**
Control 1 week	25	254.1±7.6	104.6±3.4
Diabetic 1 week	24	189.9±9.9***	401.7±25.5***
Control 4 weeks	33	341.9±11.8	91.1±1.7
Diabetic 4 weeks	25	225.8±7.8***	437.9±11.9***
Control 12 weeks	36	363.9±14.9	96.7±3.9
Diabetic 12 weeks	30	237.3±11.2***	479.1±8.8***

Animals were weighed and blood glucose levels were measured before sacrifice. The triple asterisk indicates a p<0.001, significantly different from control, using the two-tailed Student’s *t*-test.

The effect of age on the expression of ionotropic glutamate receptor subunits was not the aim of this study. Gels were not loaded with samples obtained from animals of different age.

### Effect of diabetes on protein expression of AMPA receptor subunits

**Figure 1:**
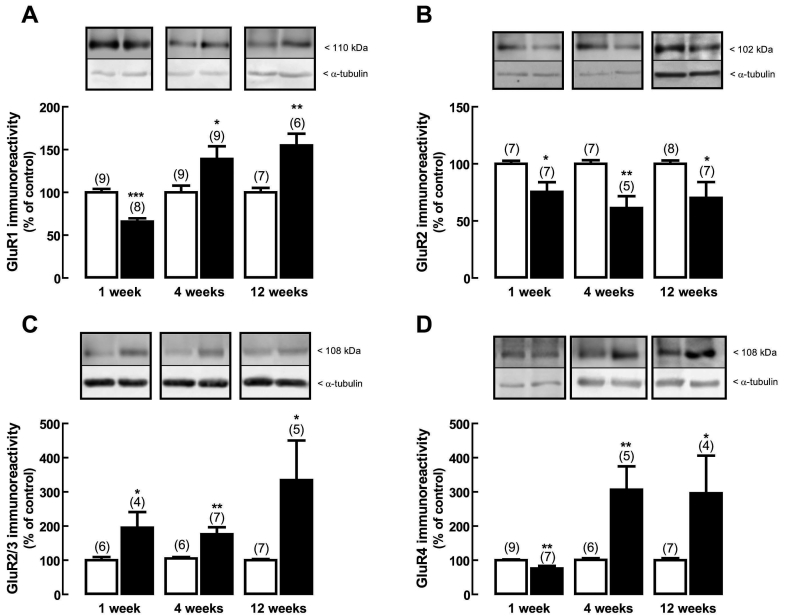
Effect of diabetes on the protein levels of AMPA receptor subunits. Total retinal extracts were obtained from rat retinas with diabetes for one, four, and 12 weeks (black bars) and from age-matched control rats (white bars). Extracts were assayed for (**A**) GluR1, (**B**) GluR2, (**C**) GluR2/3, and (**D**) GluR4 subunits immunoreactivity by western blot analysis. Representative western blots are presented above the bars for each time point tested. The densitometry of each band was analyzed. The results are expressed as percentage of age-matched controls and are presented as the mean±SEM, for the indicated number of animals. In each western blot analysis, a reprobing for detection of α-tubulin was performed to confirm that similar amounts of protein were applied to the gel. The asterisk indicates a p<0.05, the double asterisk indicates a p<0.01, and the triple asterisk indicates a p<0.001, significantly different from control, using the two-tailed Student’s *t*-test.

The effect of diabetes on the rat retinal protein levels of GluR1, GluR2, GluR2/3, and GluR4 receptor subunits was examined by western blot analysis. One week after the onset of diabetes, the protein levels of GluR1 (Figure 1A) significantly decreased to 66±4%, compared to the protein levels in age-matched controls. At four and 12 weeks after the onset of diabetes, the protein levels of GluR1 subunit significantly increased to 139±15% and 154±14% of the control, respectively. The protein levels of GluR2 subunit (Figure 1B) significantly decreased to 75±8% of the control after one week of diabetes, to 61±10% of the control after four weeks of diabetes, and to 70±14% of the control after 12 weeks of diabetes. The protein levels of GluR2/3 subunits increased after one, four, and 12 weeks of diabetes to 195±46%, 176±20%, and 334±116% of the control, respectively (Figure 1C). The protein levels of GluR4 subunit (Figure 1D) significantly decreased in one week diabetic rat retinas to 76±8%, as compared to age-matched controls, but the levels increased after four and 12 weeks of diabetes to 307±68% and 296±110% of the control, respectively.

### Effect of diabetes on AMPA receptor subunits gene expression

**Figure 2:**
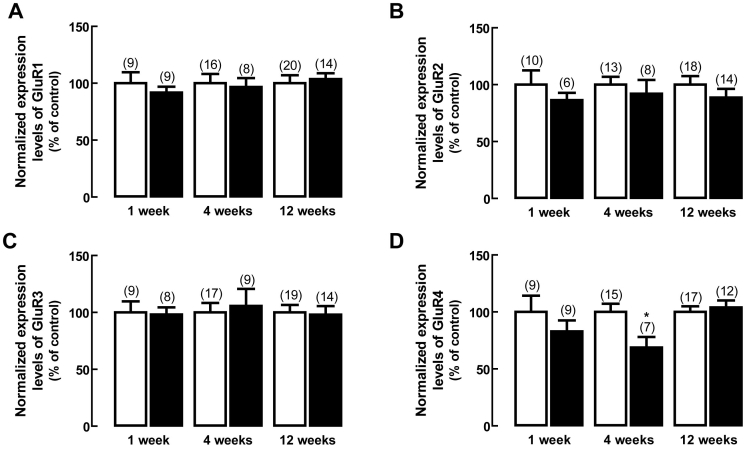
Effect of diabetes on the transcript levels of AMPA receptor subunits. Total RNA was isolated from rat retinas with one, four and 12 weeks of diabetes (black bars) and from rat retinas of age-matched controls (white bars). The transcript levels of (**A**) GluR1, (**B**) GluR2, (**C**) GluR3, (**D**) GluR4 subunits were analyzed by qPCR. The results represent the normalized expression levels for each subunit, as explained in Methods, and are presented as the mean±SEM, for the indicated number of animals. The asterisk indicates a p<0.05, significantly different from control, using the two-tailed Student’s *t*-test.

The effect of diabetes on AMPA receptor subunit-encoding transcripts in the rat retina was analyzed by qPCR (Figure 2). The transcript levels of splice variants at the C-terminal (short and long) for GluR2 and GluR4 subunits, and the flip and flop splice variants for GluR1–4 subunits were also evaluated. Compared to the transcript levels in the retina of age-matched control rats, there were no significant changes in GluR1 (Figure 2A), GluR2 (Figure 2B), GluR3 (Figure 2C), and GluR4 (Figure 2D) subunits expression, except for GluR4 at the four weeks time point, which decreased to 68±9% of the control.

For GluR2 and GluR4 subunits alternative splicing occurs at the C-terminal region [34-36]. The GluR2-short splice variant was much more abundant than the long splice variant in all samples (Table 4). Diabetes did not cause significant changes in the GluR2-short/-long ratio, except for the four weeks time point (Table 4). The GluR4-long splice variant was more abundant than the short splice variant in all samples, but no changes were found in the diabetic groups compared to age-matched controls (Table 4).

**Table 4:** Ratios of the flip:flop and short:long splice variants for AMPA receptor subunits

**AMPA receptor splice variants**	**1 week**		**4 weeks**		**12 weeks**	
**Control**	**Diabetic**	**Control**	**Diabetic**	**Control**	**Diabetic**
GluR1 flip:flop	1.19±0.05	1.24±0.04	1.08±0.02	1.06±0.02	1.30±0.05	1.08±0.03**
GluR2-short:long	37.7±5.0	40.5±3.4	33.6±1.5	54.2±9.1**	37.6±2.2	43.9±2.3
GluR2 flip:flop	2.69±0.23	2.43±0.13	2.54±0.24	2.71±0.11	2.49±0.12	2.85±0.16
GluR3 flip:flop	1.01±0.06	1.01±0.05	1.13±0.09	1.14±0.10	1.11±0.08	1.03±0.05
GluR4-short:long	0.72±0.08	0.68±0.04	0.61±0.04	0.61±0.02	0.75±0.05	0.71±0.05
GluR4 flip:flop	0.40±0.04	0.44±0.03	0.51±0.06	0.50±0.08	0.54±0.07	0.63±0.08

The ratio is calculated using the normalized expression levels for each isoform for each animal and expressed as the mean±SEM. The double asterisk indicates a p<0.01, and the triple asterisk indicates a p<0.001, significantly different from control, using the two-tailed Student’s *t*-test.

For all AMPA receptor subunits, so-called flip and flop versions are present [37]. Receptors containing flip subunits exhibit significantly slower desensitization kinetics and a greater steady-state component in their response to glutamate relatively to those containing flop subunits [37-40]. Overall, no significant changes were observed in flip/flop ratio due to diabetes (Table 4).

### Effect of diabetes on protein and gene expression of kainate receptor subunits

**Figure 3:**
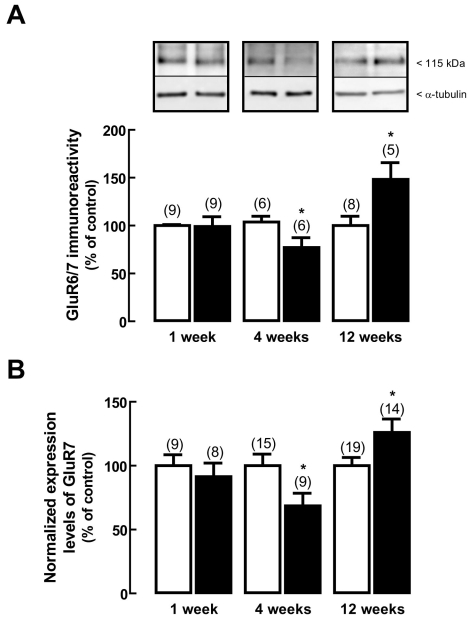
Effect of diabetes on kainate receptor subunits expression. Total protein extracts (**A**) and total RNA extracts (**B**) were prepared from rat retinas with one, four, and 12 weeks diabetic rat retinas (black bars) and from age-matched controls (white bars). **A:** Total retinal extracts were assayed for GluR6/7 subunits immunoreactivity by western blot analysis. Representative western blots are presented above the graph. The densitometry of each band was analyzed. The results are expressed as percentage of age-matched controls and are presented as the mean±SEM, for the indicated number of animals. In each western blot analysis, a reprobing for detection of α-tubulin was performed to confirm that similar amounts of protein were applied to the gel. The asterisk indicates a p<0.05, significantly different from control, using the two-tailed Student’s *t*-test. **B:** The transcript levels of GluR7 subunit were analyzed by qPCR. The results represent the normalized expression levels for GluR7 subunit, as explained in Methods, and are presented as the mean±SEM, for the indicated number of animals. The asterisk indicates a p<0.05, significantly different from control, using the two-tailed Student’s *t*-test.

One week after the onset of diabetes, there were no changes in the protein levels of GluR6/7 subunits (Figure 3A). After four weeks of diabetes, the protein levels of GluR6/7 significantly decreased to 77±10%, when compared to age-matched controls. However, after 12 weeks of diabetes, the protein levels of GluR6/7 subunits significantly increased to 148±18% of the control.

The transcript levels of GluR7 subunit (Figure 3B) were unchanged in the rat retinas, one week after the induction of diabetes, as compared to age-matched controls. However, the transcript levels of this subunit significantly decreased to 69±10% of the control after four weeks of diabetes, and increased after 12 weeks to 126±10% of the control.

The transcript levels of GluR5 and GluR6 subunits were below the detection level (data not shown).

### Effect of diabetes on protein and gene expression of NMDA receptor subunits

**Figure 4:**
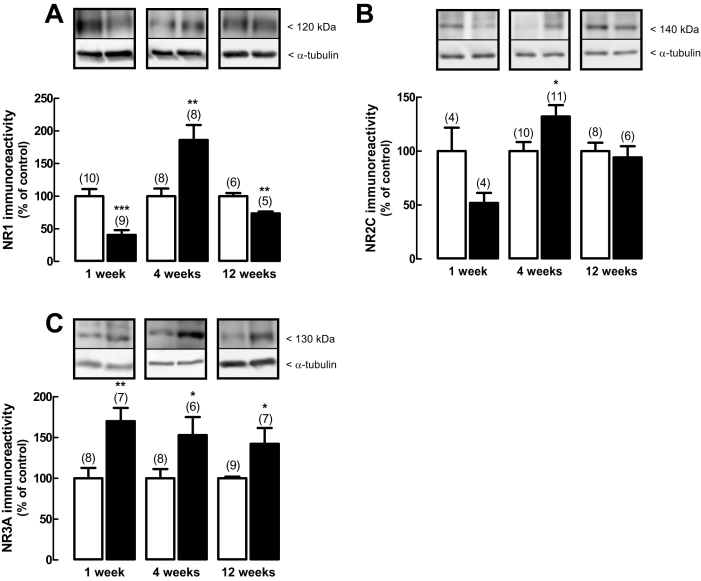
Effect of diabetes on the protein levels of NMDA receptor subunits. Total retinal extracts were prepared from rat retinas with one, four, and 12 weeks of diabetes (black bars) and from age-matched control rats (white bars). Extracts were assayed for (**A**) NR1, (**B**) NR2C, and (**C**) NR3A subunits immunoreactivity by western blot analysis. Representative western blots are presented above the graphs for each time point tested. The densitometry of each band was analyzed. The results are expressed as percentage of age-matched controls and are presented as the mean±SEM, for the indicated number of animals. In each western blot analysis, a reprobing for detection of α-tubulin was performed to confirm that similar amounts of protein were applied to the gel. The asterisk indicates a p<0.05, the double asterisk indicates a p<0.01, and the triple asterisk indicates a p<0.001, significantly different from control, using the two-tailed Student’s *t*-test.

Diabetes induced changes in the NR1 subunit protein levels in the retina (Figure 4A). In retinas from rats who had diabetes one week, the protein expression levels of NR1 subunit significantly decreased to 41±7% of the control. However, after four weeks of diabetes, the protein levels of NR1 subunit significantly increased to 186±23% of the control, and 12 weeks after the onset of diabetes, the protein levels of NR1 subunit decreased to 74±3% of the control.

The protein expression of NR2C (Figure 4B) was not significantly changed in retinas of rats with diabetes for one week, compared to age-matched controls; it significantly increased to 132±10% of the control after four weeks of diabetes. In rats who had 12 weeks of diabetes, the protein levels of NR2C subunit were similar to the control. The protein expression levels of NR3A subunit (Figure 4C) increased in the retinas of rats after one week of diabetes to 170±16% of the control, to 153±22% of the control after four weeks of diabetes, and to 142±19% of the control after 12 weeks of diabetes.

**Figure 5:**
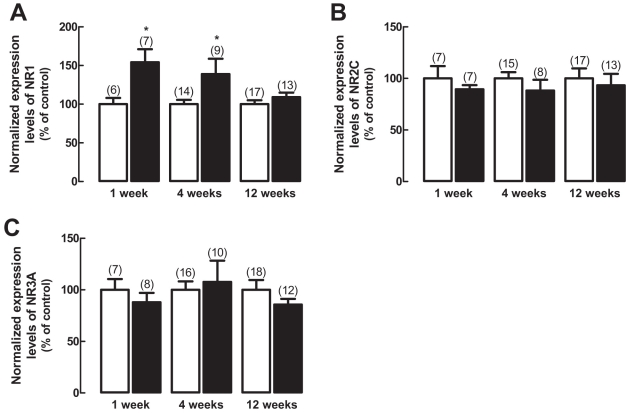
Effect of diabetes on the transcript levels of NMDA receptor subunits. Total RNA extracts were prepared from rat retinas with one, four, and 12 weeks of diabetes (black bars) and from age-matched controls (white bars), and the transcript levels of (**A**) NR1, (**B**) NR2C, and (**C**) NR3A subunits were analyzed by qPCR. The results represent the normalized expression levels for each subunit, as explained in Methods, and are presented as the mean±SEM, for the indicated number of animals. The asterisk indicates a p<0.05, significantly different from control, using the two-tailed Student’s *t*-test.

The transcript levels of NR1 subunit (Figure 5A) were upregulated after one and four weeks of diabetes, as compared to age-matched controls, but were unchanged after 12 weeks. The transcript levels of NR2C (Figure 5B) and NR3A (Figure 5C) subunits remained unchanged at the three time points investigated.

## Discussion

There is evidence to support the involvement of glutamate in the pathogenesis of DR. In the retina of diabetic animals, the metabolism of glutamate is impaired [24,25] and the release of d-aspartate is increased [28]. Furthermore, in cultured retinal neural cells we previously found that elevated glucose changes the content of AMPA and kainate glutamate receptor subunits [29]. These findings led us to hypothesize that ionotropic glutamate receptor subunits expression is changed in the retina of diabetic rats. Changes in subunit composition may alter glutamate receptor properties, therefore affecting retinal neurotransmission and consequently vision. Thus, the main purpose of this study was to investigate whether diabetes changes the expression of ionotropic glutamate receptor subunits in the retina, and to determine if changes at the protein level were associated with changes at the gene expression level.

Our results demonstrate that diabetes does alter the expression of ionotropic glutamate receptor subunits in the retina, but mainly at the protein level. Of particular interest is the significant downregulation of GluR2 subunit protein expression, found at all three time points studied. AMPA receptors are usually less permeable to calcium than the NMDA receptor. However, AMPA receptors lacking the GluR2 subunit are calcium-permeable [41-43]. The downregulation of GluR2 subunit may serve as a molecular switch leading to the formation of calcium-permeable AMPA receptors, which may enhance the toxicity of endogenous glutamate following a neurologic insult [44]. Previously, it was reported that the calcium-binding proteins, calbindin and parvalbumin, are increased in the retina of STZ-induced diabetic rats, probably to increase the buffering capacity of retinal cells to maintain the calcium homeostasis and protect them against the damaging effects of excessive calcium influx during overstimulation of ionotropic glutamate receptors [31].

We have also found an increase in GluR2/3 subunits, suggesting that GluR3 subunit is upregulated in diabetic rat retinas. The lack of a suitable anti-GluR3 subunit antibody has prevented us from testing this possibility in a more direct way. Previously, it was demonstrated that GluR2/3 subunits are more abundant in the retinas of rats with diabetes four four and 16 weeks in ganglion, amacrine and bipolar cells as well as in the inner and outer plexiform layers [31]. The same work reported increased levels of NR1 subunit in the retinas of rats who had diabetes for four or 16 weeks. In our case, we found that NR1 subunit protein levels were not elevated throughout the complete course of diabetes; NR1 subunit protein levels were downregulated at one week, upregulated at four weeks, and downregulated at 12 weeks. This is particularly relevant since most of the studies deal with only one time point of diabetes and, as we found, the diabetic rat retina is not a static tissue.

Another important finding of this study is the increase in NR3A subunit in the retinas of diabetic animals. In vitro studies indicate that NR3A is a modulatory subunit that can alter NMDA receptor activity and function [45-48]. Electrophysiological studies have shown that the NR3A subunit can co-assemble with NR1 and NR2A to form functional NMDA receptors with decreased NMDA receptor activity and decreased Ca^2+^ flux [47,48]; mice lacking the NR3A subunit show enhanced NMDA receptor activity [46]. There is no data demonstrating that NR3A preferentially assembles with certain NMDA receptor subunits. However, in NR3A-overexpressing transgenic mice, NR3A is incorporated into at least a subset of NMDA receptors, mitigating their responses in hippocampal neurons [49]. Considering its suppressive effects on NMDA receptors, NR3A has been suggested to act as a neuroprotective modulator. NR3A subunit has been found in the inner retina from the rodent retina from an early postnatal age and persisting into adulthood [50]. The same study reported that NR3A may modulate NMDA receptor-mediated calcium influx in retinal ganglion cells and amacrine cells, decreasing the intracellular calcium changes concentration. We have previously reported an increase in glutamate release during diabetes [28], which may lead to glutamate excitotoxicity. The increase in NR3A subunit in diabetic rat retinas may be a compensatory mechanism to prevent calcium overload.

Previously, in cultured retinal neural cells, we found that the protein levels of GluR1 and GluR6/7 subunits decrease and GluR2 and KA2 subunits increase [29]. Therefore, the data obtained in this work does not completely fit with the in vitro study, where we investigated only one time point of elevated glucose incubation (seven days). Also, in diabetic human postmortem retinas we have found that GluR2 and NR1 subunits are altered, mainly at the plexiform layers and ganglion cell layer [30]. Thus, the main conclusion of this work is that hyperglycemia induces alterations in ionotropic glutamate receptors, which may have consequences in receptor function and eventually in retinal cell viability.

Our results also show that in general there was no correlation between the results found for protein and mRNA expression levels, indicating that the changes observed in the ionotropic glutamate receptor subunits at the protein level are not the result of changes at the transcript level. In this work, the data are derived from the total retina, giving an average of the expression level of a particular ionotropic glutamate receptor subunit in the whole retina, losing the cell-to-cell variation. The level of expression of each glutamate receptor subunit is determined, at any particular time, by the balance of the rates of gene transcription, mRNA translation, mRNA degradation, and protein degradation. The most significant changes are observed at the protein level, which suggests that diabetes changes the ratio of protein synthesis as well as degradation. Recently, it was reported that genes for protein synthesis are upregulated in the retina of diabetic mice [51]. At least in the STZ-induced diabetic rat model, the presumption that changes at the mRNA expression are translated to similar changes at the protein level should not be followed.

This work also addressed whether diabetes induces alterations in the splicing mechanisms encoding for different C-terminal variants of GluR2 and GluR4 and on the splicing machinery of AMPA receptor subunits encoding for flip and flop isoforms. In control retinas, the short splice variant of GluR2 is more abundant than the long splice variant, and GluR4-long is also more abundant than GluR4-short, in accordance with previous reports [32]. Our data did not provide clear evidence for profound alterations in either the ratio of the long and short variants or the ratio of flip and flop splice variants, suggesting that the splicing mechanisms leading to these isoforms are not altered by diabetes.

In conclusion, the observed changes in ionotropic glutamate receptor subunits content show that diabetes affects a main component of the excitatory neurotransmission, suggesting potential alterations in synaptic communication throughout the retina, which may contribute to color vision defects or loss of contrast sensitivity [5,52]. Also, the expression of ionotropic glutamate receptors with abnormal subunit composition may cause neurons to become more vulnerable to excitotoxic stress. It remains to be determined whether these changes contribute to the subsequent neurodegeneration found in diabetic retinas.
